# Management of acute maxillary sinusitis after sinus bone grafting procedures with simultaneous dental implants placement – a retrospective study

**DOI:** 10.1186/s12879-016-1398-1

**Published:** 2016-03-08

**Authors:** Lucian Chirilă, Cristian Rotaru, Iulian Filipov, Mihai Săndulescu

**Affiliations:** “Dan Theodorescu” Clinical Hospital of Oral and Maxillo-Facial Surgery, Bucharest, Romania; Department of Oral and Maxillo-Facial Surgery, Faculty of Dental Medicine, Carol Davila University of Medicine and Pharmacy, Bucharest, Romania; Dental Concept Studio, Bucharest, Romania; Opera Dental, Bucharest, Romania; MINEC, Bucharest, Romania; Department of Oral Implantology, Faculty of Dental Medicine, Carol Davila University of Medicine and Pharmacy, Bucharest, Romania

## Abstract

**Background:**

The sinus lift was first described in 1974 and it has proven to be a predictable procedure ever since. The complications of this surgical procedure are reported in the literature to be low, and can include acute maxillary sinusitis, scattering of the grafting material into the sinus cavity, wound dehiscence and Schneiderian membrane perforations. We aimed to evaluate the rate of acute maxillary sinusitis after sinus lift procedures and the appropriate management strategies.

**Methods:**

Between 2013 and 2015, 245 dental implants were placed in 116 patients (76 males and 40 females) with concomitant bone augmentation of the maxillary sinus floor. The sinus lifting procedure was bilateral in 35 patients and unilateral in 81 patients (a total of 151 sinuses).

**Results:**

Maxillary sinusitis occurred in 5 patients (4.3 %). The clinical signs of infection were: headache, locoregional pain, cacosmia, inflammation of the oral buccal mucosa and rhinorrhea or unilateral nasal discharge. A mucosal fistula was observed during inspection in one patient. The management included only the removal of the grafting material in 3 patients, in 1 patient the grafting material was removed together with all the implants, and in 1 patient only 2 implants and the grafting material were removed, 1 implant being left in place. The sinus cavity was irrigated with metronidazole solution and antibiotic therapy with clindamycin and metronidazole was prescribed for 10 days. Subsequently, all signs of infection disappeared within 5 to 7 days and normal sinus function and drainage were restored.

**Conclusions:**

Although sinus lift is regarded as a safe and reliable procedure, acute sinusitis is a possible complication which has to be managed immediately in order to reduce the risk of further complications like pansinusitis, osteomyelitis of the maxillary bone, and spreading of the infection in the infratemporal space or orbital cavity. To minimize risk, caution must be taken with all the steps of the procedure, in order not to obliterate the ostium, impairing maxillary sinus clearance.

## Background

Ever since it was first performed in 1974, and later published in 1986 [1], the maxillary sinus bone grafting or sinus lift procedure has proven to be a predictable and relatively safe procedure [2–4]. This surgical technique allows the reconstruction of the atrophic posterior maxilla, in order to replace the missing posterior maxillary teeth with implant-supported restorations.

The complications of this surgical procedure are reported in the literature to be low, and can include acute maxillary sinusitis [5], wound dehiscence [6], and Schneiderian membrane perforations [7], with consecutive scattering of the grafting material in the sinus cavity [8].

In the present study we aimed to evaluate the incidence of one of the late complications of the sinus lift procedure – acute maxillary sinusitis – and its appropriate management strategies. Since sufficient residual bone height has been shown to successfully allow the immediate placement of dental implants at the same time with the sinus bone augmentation procedure [9], we focused only on the one stage method – lateral window sinus lift with concomitant insertion of the implants.

## Methods

We performed a retrospective study on the patients which underwent surgical procedures of sinus bone grafting (lateral window technique), with simultaneous dental implant placement in three medical centers in Bucharest, Romania between January 2013 and January 2015. The inclusion criteria were based on the planned surgical procedure: we consecutively included patients which at the time of surgery presented between 3 and 5 mm of bone height between the alveolar crest and the maxillary sinus floor and a minimum of 6 mm width in bucco-oral direction, in order to allow the immediate placement of the dental implants, but still requiring a lateral window surgical procedure. Written informed consent for the surgical procedure and for the use of the data (including radiographs and intraoral pictures) for research, publication and teaching purposes was obtained from all patients.

### Patient selection

All patients were preoperatively assessed, determining both the dental and the general health status. A thorough dental treatment plan was discussed with each patient, and the restoration of the missing teeth by implant-retained fixed prosthesis was decided. A cone-beam computed tomography (CBCT) was performed in each case, in order to determine the exact available bone volume. Patients with less than 3 mm of bone height under the maxillary sinus floor required a staged approach – an initial sinus bone grafting procedure, with the placement of the dental implants 6 to 9 months later – and were excluded from this study. Patients with more than 5 mm of bone height below the maxillary sinus floor were scheduled to receive dental implants with a minimally-invasive crestal approach sinus bone grafting, and were also excluded from the present study.

### Surgical protocol

Before scheduling the dental implant procedure, all patients underwent the treatment of all acute and chronic dental conditions – dental caries, root canal treatments, extraction of unrestorable roots – and a thorough dental cleaning.

The surgical procedure was initiated with the disinfection of the surgical site and the perioral tissues using a povidone iodine solution (Betadine, Egis Pharmaceuticals PLC, Budapest, Hungary). Local anesthesia was applied using articaine 4 % solution with adrenaline 1:200000 (Ubistesin, 3 M-Espe, St Paul, MN, USA). A crestal incision was performed, followed by a vertical releasing incision in the canine area and a distal vertical incision in the second or third molar area, in order to allow the reflection of a full thickness flap to expose the anterolateral wall of the maxillary bone. At this level a square or rounded shape osteotomy was done using low speed burrs, with abundant sterile saline irrigation, in order to gain access to the maxillary sinus. Using special instruments, the sinus membrane was carefully elevated from the floor and from the anterolateral and the medial walls of the maxillary sinus. After the elevation of the Schneiderian membrane, the dental implant sites were prepared using low speed calibrated burrs, specific to the implant system used. The bone grafting material was placed under the elevated sinus membrane, and the dental implants were inserted with a good primary stability, at a torque value varying from 30 to 50 Ncm.

**Fig. 1:**
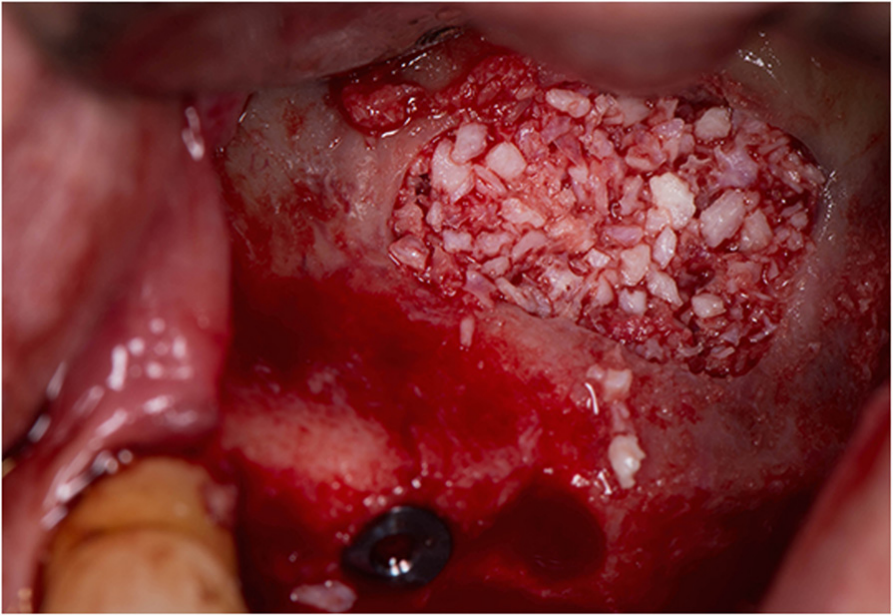
Lateral approach sinus bone graft, with simultaneous placement of two dental implants. The grafting material used is a combination of human allograft and autologous bone chips

Depending on the volume that was required to be augmented, the grafting material used was a mix of xenograft (Cerabone, Botiss biomaterials GmbH, Gerlingen, Germany or Gen-Os, Osteobiol, Tecnoss Dental, Torino, Italy) and autologous bone chips, human allograft (Maxgraft, Botiss biomaterials GmbH) with autologous bone chips, a mix of xenograft and allograft or alloplastic grafting material (Maxresorb, Botiss biomaterials GmbH). Based on the patients’ preferences, dental implants used were either MIS (Medical Implant System, MIS Implant Technologies Ltd, Shlomi, Israel) or Megagen (MegaGen, Gyeongsan, Daegu, South Korea), with diameters varying from 3.75 to 5.5 mm and length varying from 10 to 13 mm (Fig. 1).

The periosteum at the base of the full thickness flap was dissected, in order to ensure a tension-free closure of the surgical wound, the flap was repositioned and it was sutured with horizontal mattress sutures, 5.0 thickness (Surgipro II, Covidien, Dublin, Republic of Ireland).

In some cases we administered locally a 4 mg dexamethasone intramucosal injection, to reduce the postoperative edema and subsequent discomfort, as shown in the literature [10].

Postoperatively the patients were instructed not to blow their nose, to sneeze with the mouth wide open and to avoid drinking with straws, in order not to modify the air pressure inside the maxillary sinus. They were also instructed how to perform oral hygiene in the healing period. Antibiotics – clindamycin 300 mg orally every 8 h – and non-steroid anti-inflammatories – ketoprofen 100 mg orally every 8 h – were prescribed for 5 days postoperatively.

The sutures were removed 10 to 14 days postoperatively. The patients were recalled 48 h after the intervention for a postop check, and then every 30 days, to monitor the healing process.

### Statistical analysis

For the statistical analysis we used SPSS Statistics for Windows (version 22.0, IBM Corporation, Armunk, NY, USA), with a significance value of *p* = 0.05 and a confidence interval of 95 %. For variables with normal (Gaussian) distribution, means ± standard deviation (SD) are presented, and comparisons were performed with the independent-samples *T* test. For variables with non-Gaussian distribution, median (interquartile range – IQR) are presented, and comparisons were performed with the Mann–Whitney *U* test. Sample distribution was tested using the Shapiro-Wilk or Kolmogorov-Smirnov tests. The differences in proportions were assessed with the Z-test.

## Results

The inclusion criteria were met by 116 patients, of which 76 (65.5 %) were males. The mean age ± SD were 45.5 ± 10.1 years (range 26–71).

The sinus lifting procedure was bilateral in 35 patients and unilateral in 81 patients (a total of 151 sinuses). The grafting materials used are presented in Table 1.

**Table 1:** Grafting materials used

Grafting material	Number of sinuses	Percentage
Xenograft	95	62.9
Allograft	24	15.9
Xenograft and allograft mix	27	17.9
Alloplastic	3	2.0
Xenograft and alloplastic mix	2	1.3
Total	151	100

In total there were 245 dental implants placed in relationship with the augmented maxillary sinus.

Maxillary sinusitis occurred in 5 patients (4.3 %).

The clinical signs of infection were: headache, locoregional pain, cacosmia, inflammation of the oral buccal mucosa, genian and infraorbitary tumefaction and rhinorrhea or unilateral nasal discharge. A mucosal fistula was observed during inspection in one patient. The median (IQR) time to symptom onset in patients who developed infections was 5 (4) weeks. The mean ± SD age in patients who developed infections were 41.4 ± 10.6 – not statistically different from the whole group (*p* = 0.352).

Patients which developed infections received xenografts (3 patients), xenograft + allograft mix (1 patient) and alloplastic grafts (1 patient). A significantly higher proportion of patients with alloplastic grafts developed infections (1 out of 3, 33 %) compared with xenograft (3 out of 95, 3.2 % - *p* = 0.005 – see Table 2).

**Table 2:** Statistical comparison for the incidence of infection between grafting materials

Grafting material 1	Incidence of infection for grafting material 1 n/N (%)	Grafting material 2	Incidence of infection for grafting material 2 n/N (%)	*p* value	Z-score
Xenograft	3/95 (3.2)	Allograft	0/24 (0)	0.189	0.8818
Xenograft	3/95 (3.2)	Xenograft and Allograft mix	1/27 (3.7)	0.444	−0.1405
Xenograft	3/95 (3.2)	Alloplast	1/3 (33.3)	0.005	−2.6007
Xenograft	3/95 (3.2)	Xenograft and Alloplast mix	0/2 (0)	0.397	0.2553
Allograft	0/24 (0)	Xenograft and Allograft mix	1/27 (3.7)	0.171	−0.9522
Allograft	0/24 (0)	Alloplast	1/3 (33.3)	0.002	−2.8823
Allograft	0/24 (0)	Xenograft and Alloplast mix	0/2 (0)	N/A	N/A
Xenograft and Allograft mix	1/27 (3.7)	Alloplast	1/3 (33.3)	0.026	−1.9518
Xenograft and Allograft mix	1/27 (3.7)	Xenograft and Alloplast mix	0/2 (0)	0.389	0.277
Alloplast	1/3 (33.3)	Xenograft and Alloplast mix	0/2 (0)	0.181	0.9129

N/A – not applicable

## Discussion

The rate of infection in our group was relatively low – 4.3 %. However, the clinical management of the infections following this surgical procedure is very traumatic for the patient, requiring, besides antibiotic therapy, the surgical removal of the grafting material, the infected sinus mucosa and, in some cases, the implants placed adjacent to the graft.

Of the 116 patients included in the study, acute maxillary sinusitis occurred in the following patients (chronologically):

The first infection occurred in a 53 year-old female patient, who had grade 2 obesity, hypercholesterolemia, and generalized periodontal disease. We performed a lateral approach sinus bone grafting using xenograft, with concomitant placement of three dental implants in the positions of the second premolar, first molar and second molar. There were no intraoperative incidents. After two months the patient returned accusing cacosmia, headaches, pulsating pain in the canine fossa region and unilateral congestive rhinitis. Objectively, the patient presented a fistula in the first molar region, vestibular tumefaction in the premolar and molar regions, skin erythema and unilateral nasogenian tumefaction. The grafting material was removed together with the implants, with concomitant partial removal of the sinus membrane and closure of the oroantral communication with a buccal mucoperiosteal flap. We prescribed antibiotics, anti-inflammatories and nasal decongestants.

The second infection occurred in a 27 year-old healthy male patient, who underwent a sinus lift procedure with a xenograft and allograft mix and the placement of a dental implant in the second molar position. During the surgery a sinus membrane perforation occurred, 7–8 mm long, which was closed with a collagen membrane. After three weeks the patient returned, accusing a mild pulsating pain in the molar region. Upon inspection we identified a vestibular abscess in the molar region, and we performed a second surgical intervention consisting of incision and drainage of the abscess and the removal of the grafting material. The dental implant was left in place, and we continued with daily lavage with metronidazole and iodine solution for two weeks, with favourable evolution.

**Fig. 2:**
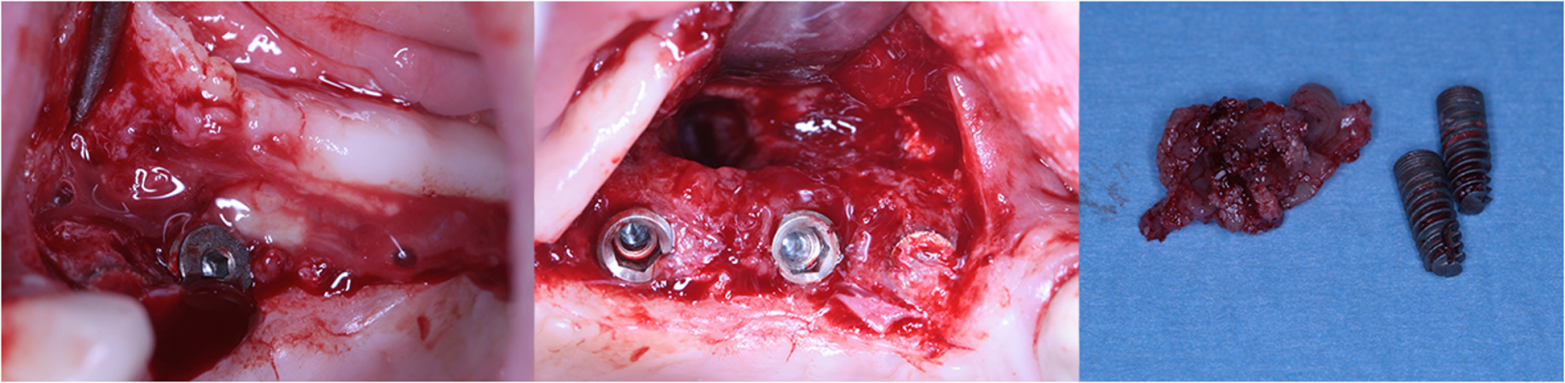
Failing sinus bone graft, following a sinus infection. The grafting material was removed, together with a fragment of the maxillary sinus mucosa and two dental implants

The third incident occurred in a 45 year-old female patient, heavy smoker (more than 20 cigarettes per day), with chronic hepatitis C virus infection and chronic obstructive pulmonary disease. Six weeks after the sinus lift surgery with alloplastic grafting material and simultaneous implant placement in the positions of teeth 2.5, 2.6 and 2.7 (#13, #14 and #15), the patient returned with odontalgia radiating in the zygomatic and periorbitary regions. We removed the grafting material together with parts of the Schneiderian membrane and two dental implants, we closed the oroantral communication and we prescribed antibiotics and anti-inflammatories (Fig. 2).

The fourth maxillary sinus infection was reported in an otherwise healthy 34 year-old female patient, 5 weeks after the uneventful placement of two dental implants replacing the missing second premolar and first molar with simultaneous lateral approach sinus lift and bone grafting with a xenograft material. The patient complained of unilateral hemicrania, and presented tumefaction of the gingival mucosa in the upper molar region and unilateral rhinitis. We removed the grafting material, we performed lavages with metronidazole and we prescribed antibiotics and anti-inflammatories, managing to maintain the two dental implants.

The last infection in our study group occurred in a 48 year-old male patient, heavy smoker (20–30 cigarettes per day), known with dyslipidemia and arterial hypertension. We performed a lateral approach sinus lift using a xenograft biomaterial and we placed two dental implants in the positions of teeth 1.6 and 1.7 (#2 and #3). There were no intraoperative incidents. Three weeks later the patient accused pain radiating in the zygomatic and periorbitary areas and congestive rhinitis. The patient presented genian and vestibular tumefaction, and we decided to remove the grafting material and the dental implants together with the infected sinus mucosa, we performed lavages with metronidazole and iodine and we prescribed antibiotics and anti-inflammatories.

All five patients recovered well after the second surgical intervention, with the remission of all symptomatology in 5 to 7 days, and normal sinus function and drainage were restored.

A PubMed search using the keywords “sinus lift” provides more than 700 results. However, adding the keyword “infection” to the previous search query narrows the results to 51. Out of these, less than half are clinical studies which discuss the complication rate of this surgical procedure. To our knowledge, there is only one other study [11] focused on evaluating the late complication rate of the sinus lift procedure, rather than the success rate of dental implants inserted in augmented sites, but it discusses both the immediate and the delayed implant insertion after the bone graft. Our results are similar to those of Vazquez et al. [11], with infectious complications in 5 out of 116 patients in our study and 9 out of 200 in theirs. This might suggest that whenever the bone volume is enough to confer a good primary stability of the implant, placing the implants at the same time with the sinus bone grafting procedure doesn’t affect the chances of success.

Our results are also in concordance with those of Ferri et al. [12], who report an infection rate of 3.5 %, but are lower than those of Khanberg et al. [13], who report signs of infection in 8 out of 36 patients, and higher than those of Kasabah et al. [14], who did not have any bone graft infection of maxillary sinuses in 146 sinus lift surgical procedures performed on 118 patients.

The abundant existing literature data on the subantral bone augmentation procedure has established it is a very safe procedure, with predictable results and a low complication rate. But as low as this complication rate may be, as we have shown in the beginning of this section, the clinical management of the complications is very difficult for the patient, with important pain and discomfort, prolonged overall treatment time and many treatment visits. The low complication rate also impacts the statistical value of this type of studies, since it is very difficult to find a statistically significant correlation between different clinical and paraclinical variables, in order to make a prediction on the chances of the occurrence of these complications. We did find a statistically significant difference when we compared the bone grafting materials used and the occurrence of the sinus infection (Table 2), but the results may be biased by the fact that we only used in three patients alloplastic bone grafting materials, and one of them developed acute maxillary sinusitis. In order to test this hypothesis, data from larger groups of patients are needed. An interesting study [15] reports a 0 % rate of infectious complications and a 100 % survival rate after two years when the implants are placed in the elevated sinus without any additional grafting material. However, the sample rate of this study [15] was low, evaluating only 47 implants inserted in 33 patients, so more studies are needed to confirm these findings.

There was no statistically significant correlation with the age of the patients, and no correlation with the smoking/non-smoking status, in accordance with the results of Levin et al. [16]. We found no correlation with intraoperative incidents like Schneiderian mucosa perforation, in accordance with another study [14], but a recent article [7] reports that graft failure was statistically higher in sinuses in which the membrane was perforated during the intervention. Also, another recent study [17] showed that certain factors such as a low albumin serum level and a prolonged intervention may constitute risk factors for complications after different oral surgeries.

A recent trend in oral rehabilitation using dental implants advocates the use of short implants, in order to avoid extensive surgical procedures like the lateral approach sinus lift [18–20]. Other studies [21] propose simplified techniques for the crestal approach sinus lift, for the same reason. Promising as these results may be, they still need to pass the trial of time, in order to replace the current standard of care in restoring the atrophic posterior maxilla.

## Conclusions

Although sinus lift is regarded as a safe and reliable technique, acute sinusitis is a possible complication which has to be managed immediately in order to reduce the risk of further complications like pansinusitis, osteomyelitis of the maxillary bone, or the spreading of the infection in the infratemporal space or orbital cavity. To minimize risk, caution must be taken with all the steps of the procedure, in order not to obliterate the ostium, impairing maxillary sinus clearance.

### Consent

The authors confirm that they have consent for publishing clinical data, treatment details and intraoral photographs.
